# Association of spinopelvic index with proximal junctional failure developing in adult spinal deformity after surgical treatment: an observational study

**DOI:** 10.1186/s12891-023-06292-2

**Published:** 2023-03-10

**Authors:** Zifang Zhang, Shang Chen, Shu Jia, Renchang Chen, Nianhu Li, Chunyang Meng

**Affiliations:** 1 Affiliated hospital of Jining Medical University, Jining Medical University, No. 89, Guhuai Road, Jining, 272007 China; 2 Orthopedic Department of Shandong University of Traditional Chinese Medicine, No. 16369, Jingshi Road, Jinan, 250014 China

**Keywords:** Adult spinal deformity, Proximal junction failure, Spinopelvic index, Long-fusion surgery

## Abstract

**Background:**

Those pelvic parameters of sacral slope (SS) and pelvic tilt (PT) correlated significantly to lumbar spine and hip joints respectively. We proposed the match between SS and PT, namely spinopelvic index (SPI), in order to investigate whether the SPI correlated to proximal junctional failure (PJF) in adult spinal deformity (ASD) after correction surgery.

**Methods:**

Ninety-nine ASD patients who had undergone long-fusion (≥ 5 vertebras) surgeries were reviewed retrospectively in two medical institutions from January 2018 to December 2019. Those SPI were calculated with the equation: SPI = SS/PT, and analyzed using the receiver operating characteristic curve (ROC) analysis. All participants were subdivided into the observational and control group. Comparisons of demographics, surgical and radiographic data between the two groups were performed. A Kaplan–Meier curve and log-rank test was used to analyze the differences in PJF-free survival time, and the 95% confidence intervals (CI) were recorded respectively.

**Results:**

Nineteen patients suffering from PJF had much smaller postoperative SPI (*P* = 0.015), but much larger TK postoperatively (*P* < 0.001). ROC analysis determined the best cutoff value of 0.82 for SPI (sensitivity = 88.5%, specificity = 57.9%; AUC = 0.719, 95%CI: 0.612–0.864; *P* = 0.003). There were 19 and 80 cases in the observational (SPI ≤ 0.82) and control group (SPI > 0.82) respectively. The incidence of PJF in the observational group was much higher (11/19 *VS* 8/80, *P* < 0.001); further logistic regression analysis showed that SPI ≤ 0.82 was associated with increased odds of PJF (odds ratio: 12.375; 95%CI: 3.851–39.771). PJF-free survival time in the observational group decreased significantly (*P* < 0.001, log-rank test), moreover, multivariate analysis demonstrated that a value of SPI ≤ 0.82 (HR 6.626, 95%CI: 1.981–12.165) was significantly associated with PJF.

**Conclusions:**

For ASD patients underwent long-fusion surgeries, the SPI should be over 0.82. The incidence of PJF may increase by 12-fold in such individuals with the immediate SPI ≤ 0.82 postoperatively.

## Background

The prevalence of adult spinal deformity (ASD), as well as the number of patients requiring a surgical procedure, is increasing. However, those deformity surgical procedures are always associated with a number of complications, and proximal junctional kyphosis (PJK) has been recognized as a major challenge over the last 20 years [1]. Proximal junctional failure (PJF) is a progressive process in the spectrum of PJK with fracture, vertebral subluxation or screws dislodgement developing in upper instrumented vertebra (UIV), UIV + 1 and/or posterior ligament complex, which may result in more serious morbidities, such as back pain and neurologic deficits [2, 3].

The significant effect of pelvis, taking full account of sacral slope (SS), pelvic tilt (PT) and pelvic incidence (PI), has been reported on keeping the full-body balance in previous studies [4, 5]. Additionally, those classifications of spinopelvic alignments were proposed according to those pelvic parameters [6, 7]. Recently, studies have been reported that full-spinal realignments in ASD patients correlated strongly to the PJK or PJF [8, 9]. Furthermore, previous studies suggested that those pelvic parameters of SS and PT correlated significantly to lumbar spine and hip joints respectively [10–13]. Therefore, we defined SS/PT as spinopelvic index (SPI) and investigated whether the abnormal SPI postoperatively could predict PJF developing after long-fusion surgeries in ASD patients?

In addition, previous studies reported that much larger global spinal alignment (thoracic kyphosis + lumbar lordosis + PI) was risk factor for PJK [3, 14]. However, Zhang et al*.* [15] suggested that the associations of thoracic kyphosis (TK) postoperatively with PJF developing may be based on the condition of hip joints. Therefore, we investigated whether the larger TK postoperatively would deteriorate the SPI, which may accelerate PJF developing in those ASD patients after correction surgery?

## Materials and methods

The study protocol was approved by the institutional review board at each site. Informed consent was obtained from all patients. We retrospectively documented the data of ASD patients in two hospitals from January 2018 to December 2019, aiming to have a minimum follow-up of 24 months. All of those patients had undergone the procedure of long-fusion (≥ 5 vertebras) with instrumentations by posterior-only approach.

General inclusion criteria for this study were as follows:

1. Age ≥ 45 years;
2. Those radiographic parameters met the criteria at least one of the followings: a, coronal curvature ≥ 20°; b, SVA ≥ 5 cm; c, PT ≥ 25°; d, TK ≥ 60° [16, 17].
3. The research data before and after surgery, including demographics, surgical and radiographic parameters, were integrated.
4. The follow-up duration ≥ 24 months.

Those having 1) prior spinal surgeries, 2) history of spinal tumor, 3) history of spinal infection such as tuberculosis, 4) ankylosing spondylitis, 5) any hip disorders, or 6) the differences between two lower extremities ≥ 2 cm were excluded.

In this current study, proximal junctional failure (PJF) was defined as fractures or subluxations happening in the UIV and/or UIV + 1; pedicle screw loosening, dislodgment, or even pullout from the UIV [18]. Demographics (age, gender, and BMI) and surgical data involving UIV, lower instrumented vertebra (LIV), and fixed segments (FS) were reviewed and documented. Postoperatively, follow-up time and PJF-free survival time after surgery were documented. Radiographs at the pre-operation, the immediate post-operation, and the final follow-up were collected.

### Radiographic evaluation

Radiographic data consisted of full-length coronal and sagittal radiographs were obtained in free- standing posture with the upper limbs resting on a support, the shoulders at 30° forward flexion, and the elbows slightly flexed [19]. All of the radiographic parameters were measured with Surgimap Software (version: 2.3.2.1; Spine Software, New York, NY).

**Fig. 1:**
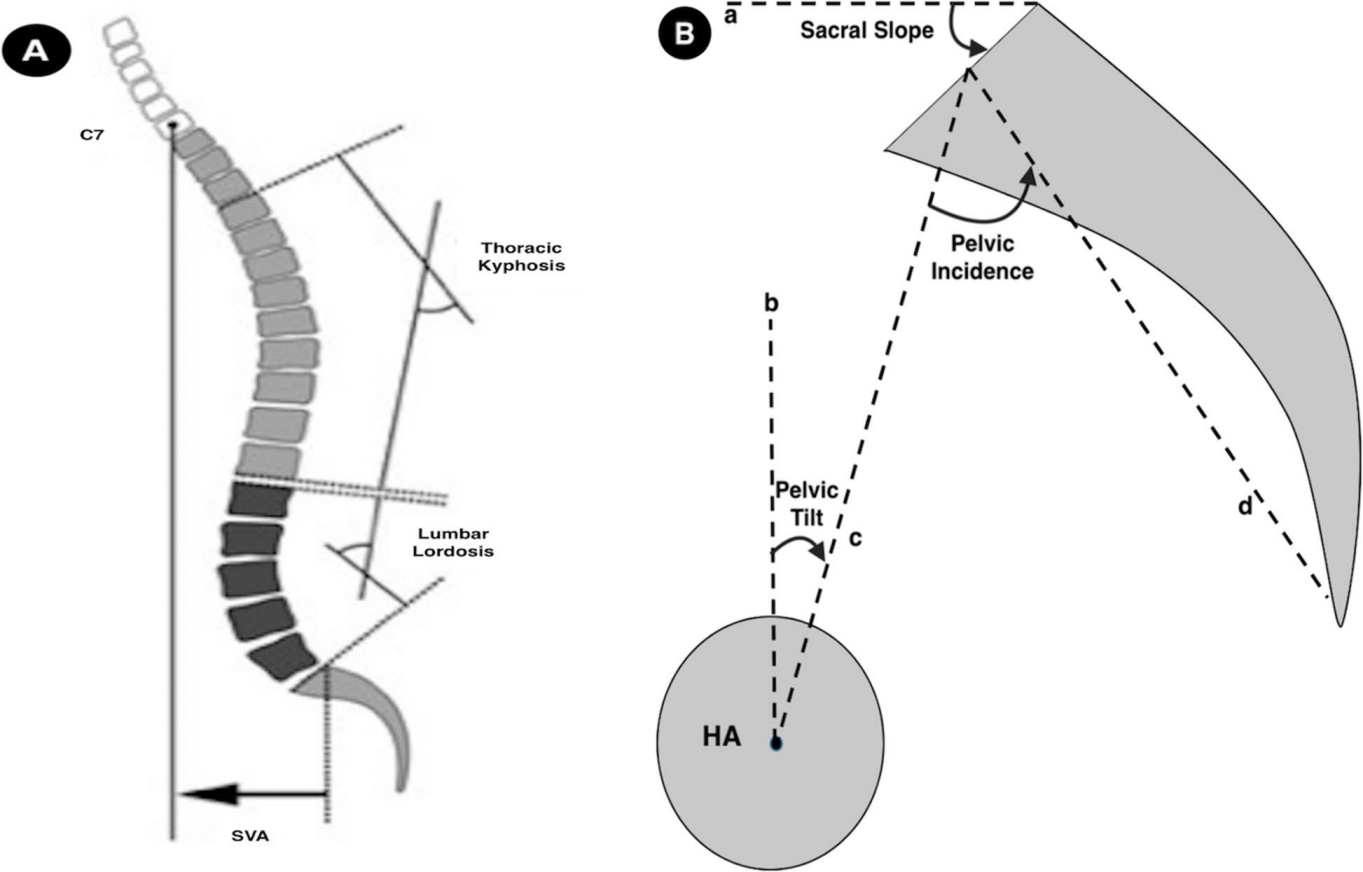
**A** Sagittal radiologic parameters: Thoracic Kyphosis (TK) measured from the superior endplate of T4 to the inferior endplate of T12 by Cobb method; Lumbar Lordosis (LL) measured from the superior endplate of L1 to the inferior endplate of S1 by Cobb method. Sagittal vertical axis (SVA) defined as the horizontal offset from the posterosuperior corner of S1 to the plumb line going through the vertebral body of C7. **B** Pelvic parameters: Sacral slope (SS): the angle between the horizontal line and the sacarl endplate; Pelvic tilt (PT): the angle between the vertical and the line through the midpoint of the sacral endplate to the femoral heads axis; Pelvic Incidence (PI): the angle between the perpendicular to the sacral plate at its midpoint and the line connecting this point to the femoral heads axis

All of the radiographic parameters concerned in this current study were shown in the Fig. 1A-B, which included thoracic kyphosis (TK), lumbar lordosis (LL), sagittal vertical axis (SVA), sacral slope (SS), pelvic tilt (PT) and pelvic incidence (PI). All of those radiographic measurements were performed by a dedicated team independent from the operating surgeons.

Kyphosis was recorded as positive value ( +), and lordosis as negative value (-). The spinopelvic index (SPI) was calculated by the equation: SPI = SS/PT.

### Statistical analysis

Continuous variables with normal distribution were expressed as the Mean ± standard deviation (SD), and abnormal data as the median. Categorical variables were expressed as counts and percentages. Firstly, comparisons between patients with and without PJF were performed. Those SPI values were analyzed using receiver operating characteristic curve (ROC), and the area under the curve (AUC) was being as the best cutoff value of SPI, by which all participants in this study were subdivided into the observational and the control group subsequently. A Kaplan–Meier curve and log-rank test were used to analyze the differences in PJF-free survival time. Multivariate analysis via a Cox proportional hazards model was applied for analyzing the risk factors of PJF-developing. Comparisons of categorical variables were analyzed with Chi-square analysis and Fisher’s exact test. Comparisons of continuous data between the observational and the control groups were performed with the Independent sample *t* test. All statistical analyses were performed using IBM SPSS statistics software (Mac version 26.0, IBM Corp.), and 95% confidence intervals were obtained; *P* < 0.05 (two-sided) was the criterion for statistical significance.

## Results

A total of 99 consecutive ASD patients (79 females and 20 males) with an average age of 64.48 years (SD 8.87; range, 45–79 year) at the surgery met the inclusion criteria. The mean time of follow-up duration was 44 months after surgery, ranging from 24 to 87 months.

In all, 19 patients (19.2%; 14 patients with fracture at the UIV or UIV + 1, and 5 patients with pedicle screw loosening, dislodgment, and/or even pullout from the UIV) developed PJF during follow-up. Comparisons of parameters at pre- and post-operation between patients with and without PJF were shown in the Table 1.

**Table 1:** Comparisons of data in patients with and without PJF

Variables	PJF group (*n* = 19)	PJF-free group (*n* = 80)	*P* Value
No. of females, n (%)	16 (84.2%)	63 (78.8%)	0.594
Age, years	66.05 ± 9.09	64.23 ± 8.19	0.394
BMI, kg/m^2^	25.58 ± 10.3	24.17 ± 10.59	0.431
Follow-up, months	51.2 ± 17.58	49.6 ± 19.38	0.72
UIV, n			0.087
T10 or above	16	51	
L2-T11	3	29	
LIV, n			0.744
S1, S2 or ilium, n	11	43	
L4 or L5, n	8	37	
FS	8.47 ± 1.93	8.21 ± 2.35	0.654
Preop-TK (°)	25.11 ± 14.05	15.23 ± 11.66	0.002^*^
Preop- LL (°)	-22.68 ± 25.1	-22.89 ± 19.2	0.970
Preop-SS (°)	14.61 ± 13.53	23.7 ± 12.43	0.006^*^
Preop-PT (°)	26.77 ± 13.1	23.39 ± 11.29	0.259
Preop-PI (°)	41.41 ± 10.88	47.21 ± 11.4	0.034^*^
Preop-SVA (mm)	56.14 ± 68.87	49.96 ± 50.59	0.659
Postop-TK (°)	31.5 ± 10.24	20.34 ± 8.89	< 0.001^*^
Postop-LL (°)	-37.95 ± 13.87	-38.43 ± 11.57	0.877
Postop-SS (°)	23.62 ± 10.76	30.83 ± 9.87	0.006^*^
Postop-PT (°)	18.88 ± 11.37	16.07 ± 8.88	0.244
postop-SPI	1.62 ± 1.88	3.66 ± 2.87	0.015^*^
Postop-SVA (mm)	13.52 ± 44.16	14.78 ± 32.07	0.886

*Note*: Mean values are presented as the mean ± SD; *, indicates *P* < 0.05; *BMI *body mass index, *UIV *Upper instrumented vertebra, *LIV *Lower instrumented vertebra, *FS *fusion segments; Preop- and postop-, pre-operative and post-operative; *TK* thoracic kyphosis, *LL* lumbar lordosis, *SS* sacral slope, *PT* pelvic tilt, *PI* pelvic incidence, *SVA* sagittal vertical axis, *SPI* spinopelvic index

**Fig. 2:**
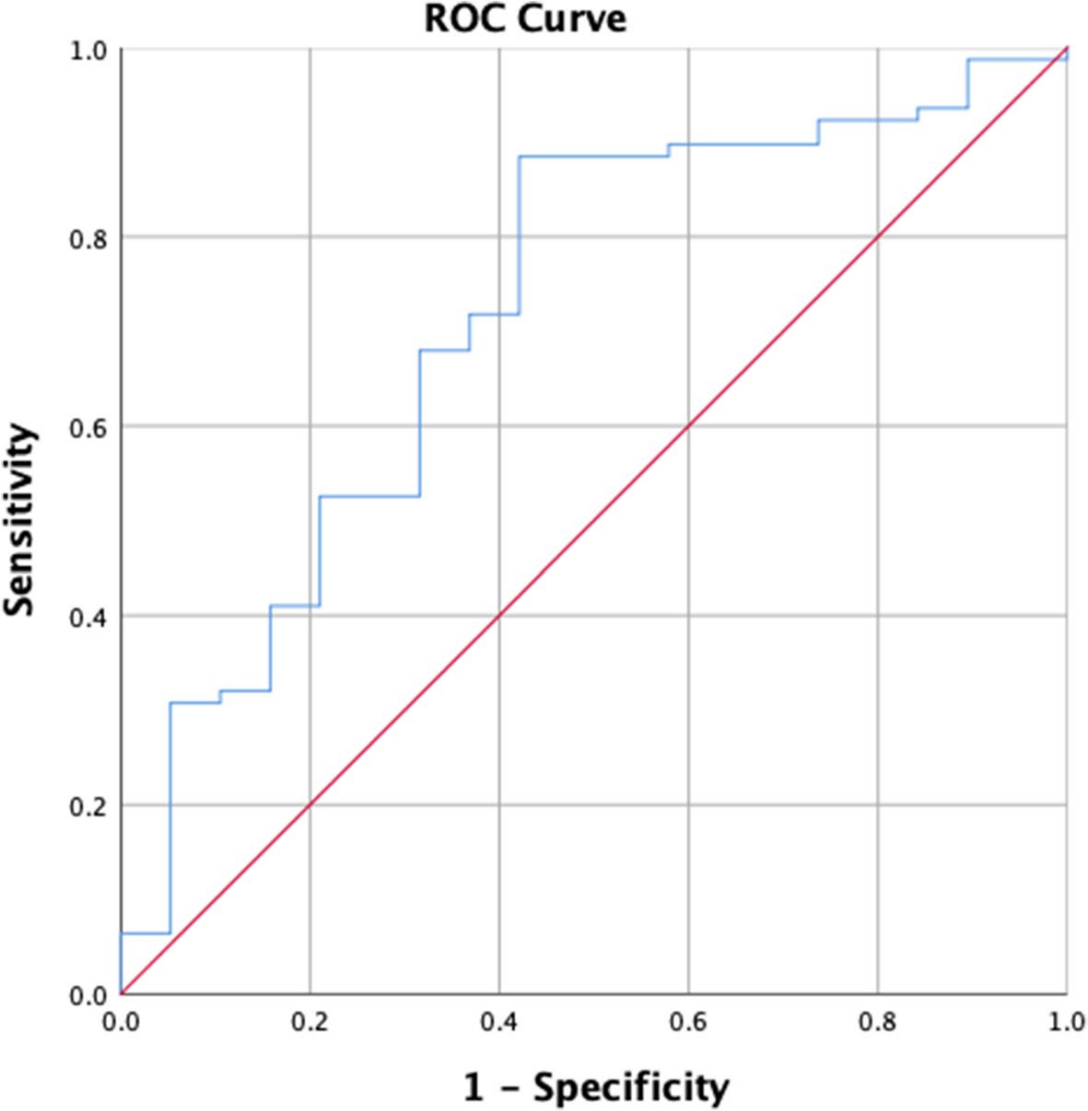
The ROC curve for predicting PJF by the spinopelvic index (SPI) value calculated by SS/PT

**Fig. 3:**
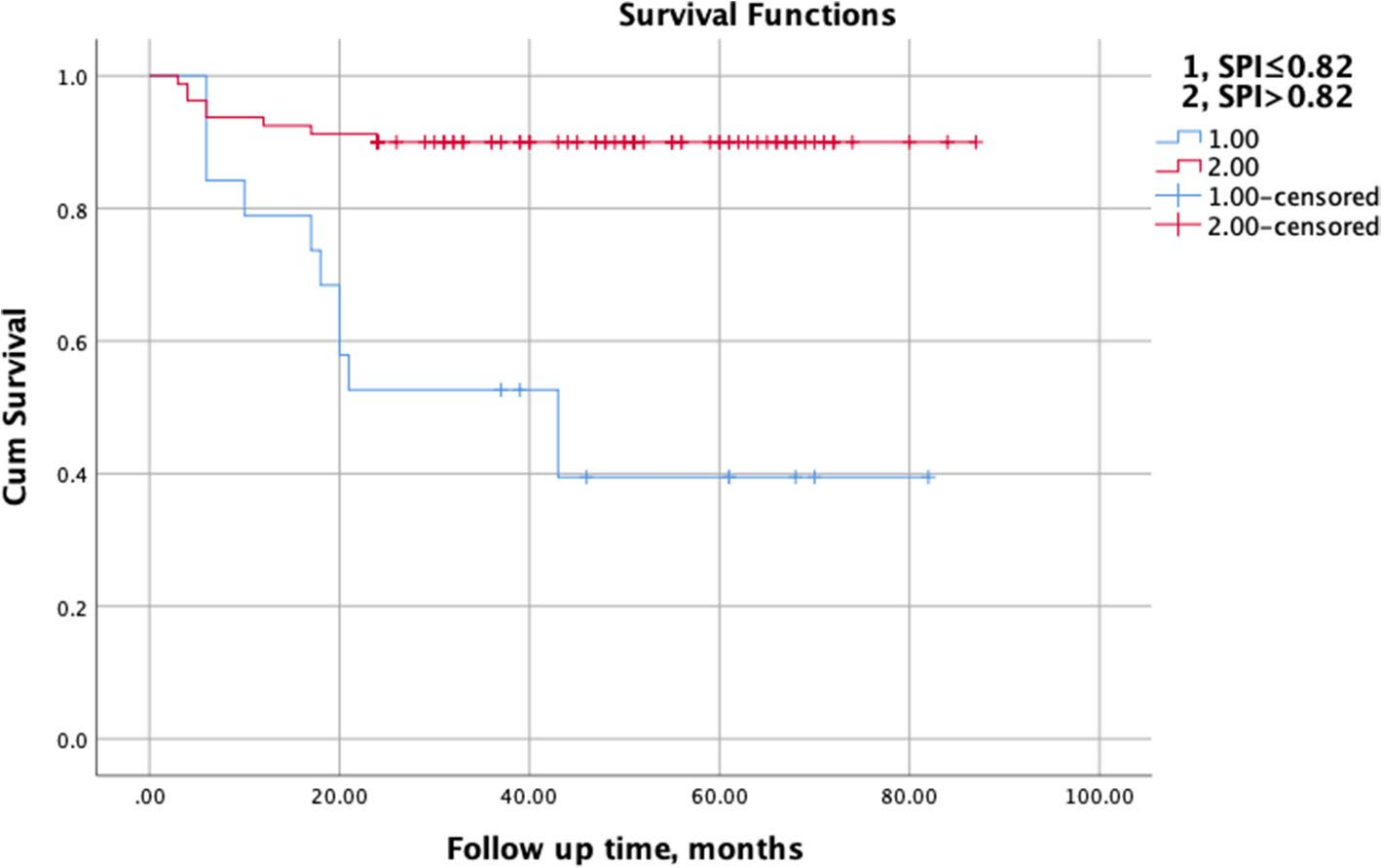
Kaplan–Meier curves illustrated the differences in PJF-free survival time stratified all patients by a threshold value of SPI (> 0.82 or ≤ 0.82)

ROC curve analysis determined an optimal threshold value of 0.82 for the SPI postoperatively (sensitivity = 88.5%, specificity = 57.9%; AUC = 0.719, 95% CI: 0.612–0.864; *P* = 0.003) (Fig. 2). There were 19 and 80 cases in the observational (SPI ≤ 0.82) and the control group (SPI > 0.82) respectively. The incidence of PJF in the observational group was much higher (11/19 *VS* 8/80, *P* < 0.001), and the OR was 12.375 (95%CI: 3.851–39.771). Furthermore, there were remarkable differences in the PJF-free survival time between the observational and the control group (*P* < 0.001, log-rank test) (Fig. 3). Comparisons of radiographic parameters, regarding to LL, SS, and PT, between the observational and control group were listed in the Table 2.

**Table 2:** Comparisons of data between observational and control group

Variable	Observational group (*n* = 19)	Control group (*n* = 80)	*P* Value
No. of females, n (%)	16 (84.2%)	63 (78.8%)	0.594
Age, years	66.26 ± 8.15	64.18 ± 8.39	0.330
BMI, kg/m^2^	24.85 ± 11.31	24.24 ± 9.59	0.685
Follow-up, month	49.46 ± 18.78	52.6 ± 20.21	0.291
UIV, n			0.087
T10 or above	16	51	
L2-T11	3	29	
LIV, n			0.744
S1, S2, or ilium	11	43	
L4-L5	8	37	
FS	8.0 ± 2.08	8.32 ± 2.32	0.578
PJF, n (%)	11 (57.9%)	8 (10%)	< 0.001 OR = 12.375
Preop-TK (°)	17.78 ± 12.12	17.02 ± 12.93	0.816
Postop-TK (°)	26.50 ± 11.33	21.53 ± 9.64	0.054
d-TK (°)	8.72 ± 12.45	4.28 ± 9.50	0.159
Preop-LL (°)	-13.0 ± 23.48	-25.25 ± 18.91	0.018^*^
Postop-LL (°)	-28.45 ± 8.54	-40.68 ± 11.50	< 0.001^*^
d-LL (°)	15.45 ± 22.53	15.43 ± 14.21	0.998
Preop-SS (°)	10.62 ± 12.19	24.68 ± 11.82	< 0.001^*^
Postop-SS (°)	17.46 ± 5.54	32.29 ± 9.17	< 0.001^*^
d-SS (°)	6.83 ± 10.47	7.70 ± 8.66	0.709
Preop-PT (°)	32.73 ± 13.56	21.93 ± 10.19	< 0.001^*^
Postop-PT (°)	25.99 ± 9.58	14.39 ± 7.92	< 0.001^*^
d-PT (°)	-6.74 ± 9.78	-7.60 ± 7.73	0.681
PI (°)	43.38 ± 11.07	46.73 ± 11.56	0.256
Preop-SVA (mm)	81.76 ± 67.36	43.72 ± 48.24	< 0.001^*^
Postop-SVA (mm)	10.5 ± 36.65	15.5 ± 34.09	0.572
d-SVA (mm)	-71.26 ± 53.81	-28.15 ± 46.82	< 0.001^*^
Postop-SPI	0.13 ± 2.12	3.77 ± 4.60	< 0.001^*^

*Note*: Mean values are presented as the mean ± SD; *, indicates *P* < 0.05; *BMI* body mass index, *UIV* Upper instrumented vertebra, *LIV* Lower instrumented vertebra, *FS* fusion segments, *PJF* proximal junctional failure, *Preop- and postop-* pre-operative and post-operative, d- the correction of radiographic parameters, *TK* thoracic kyphosis, *LL* lumbar lordosis, *SS* sacral slope, *PT* pelvic tilt, *PI* pelvic incidence, *SVA* sagittal vertical axis, *OR* odds ratio

**Fig. 4:**
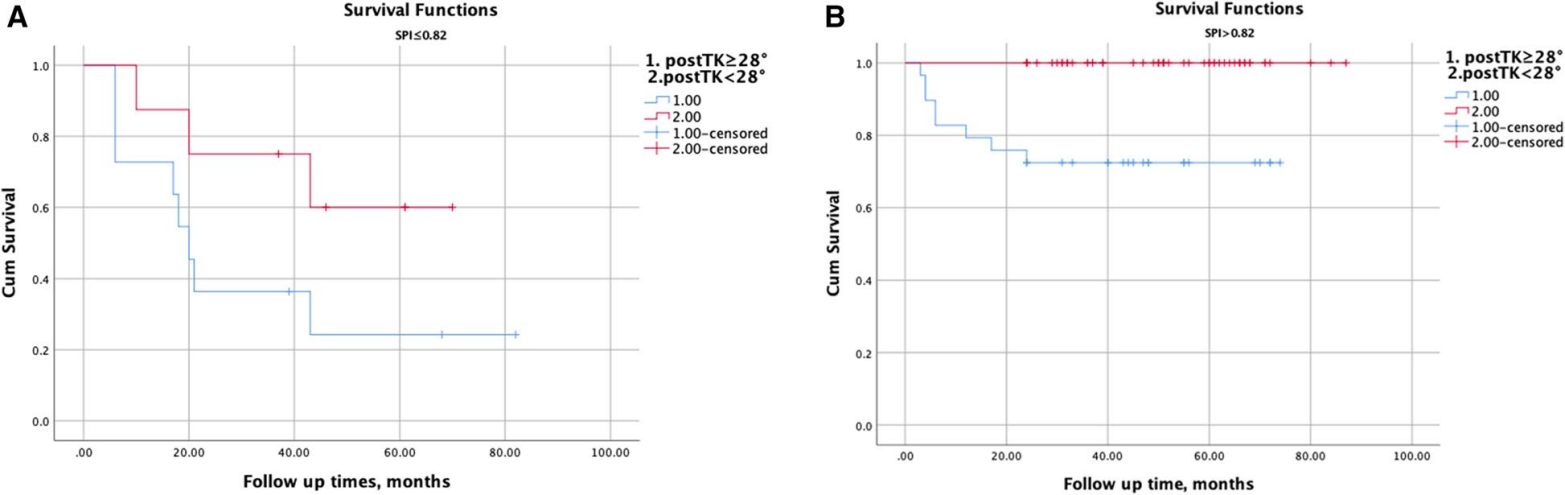
Under the condition of SPI ≤ 0.82, the PJF-free survival time showed no difference between patients with TK ≥ 28° and those with TK < 28° (**A**); For patients with SPI > 0.82, the PJF-free survival time in those having TK ≥ 28° decreased significantly (**B**)

We subdivided all patients into two groups by 28° of the immediate TK postoperatively (post-TK) suggested by professor Zhang et al. [15]. For those patients in the observational group, there was no difference in PJF-free survival time between those with post-TK ≥ 28° and those with post-TK < 28° (*P* = 0.1, log-rank test) (Fig. 4A). However, for the patients in the control group, the PJF-free survival time in those with post-TK ≥ 28° decreased significantly (*P* < 0.001, log-rank test) (Fig. 4B). Entering those radiographic parameters, having statistical differences between patients with and without PJF, into multivariate analysis via a Cox proportional hazards model, a value of SPI ≤ 0.82 (HR 6.626, 95%CI: 1.981– 12.165) and post-TK ≥ 28° (HR 1.137, 95%CI: 1.065–1.214) were significantly associated with PJF (Table 3).

**Table 3:** Multivariate analysis via a Cox proportional hazards model

Variable	HR	95% CI	*P* Value
SPI ≤ 0.82	6.626	1.981 – 12.165	0.002^*^
Postop-TK > 28° [14]	1.137	1.065 – 1.214	< 0.001^*^

*Note*: *, indicates *P* < 0.05; *SPI* spinopelvic index, *postop-TK* post-operative thoracic kyphosis, *HR* hazard ratio, *CI* confidence interval.

**Fig. 5:**
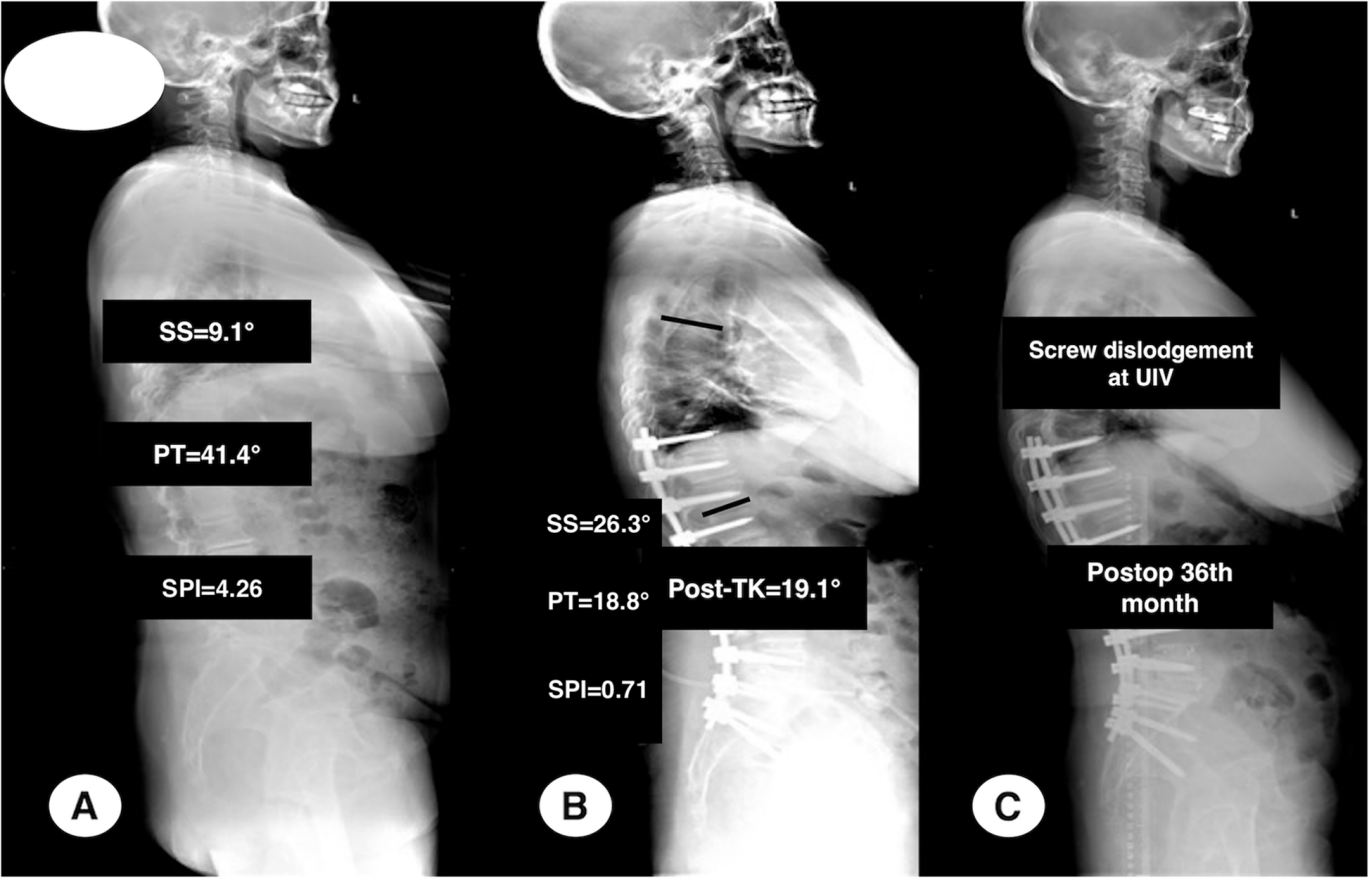
A female ASD patient with 58 years old at surgery belonging to the control group had SS of 9.1°, PT of 41.4°, and SPI of 4.26 at pre-operation (**A**); the immediate TK, SS and PT postoperatively was 19.1°, 26.3° and 18.8° respectively, and the SPI was 0.71 (**B**). The patient developed PJF with screw dislodgement of UIV at the 36^th^ month follow- up duration

**Fig. 6:**
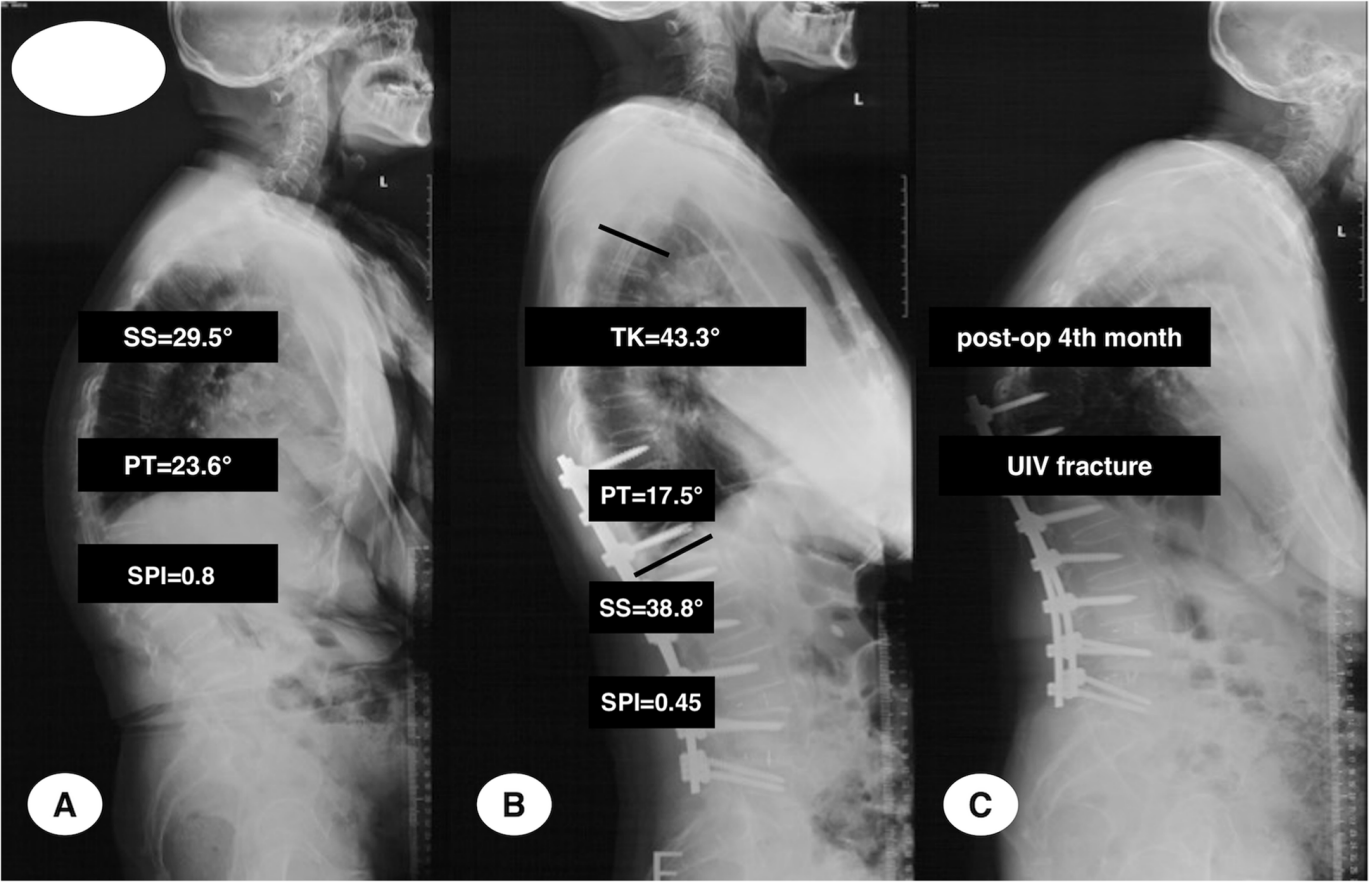
A female ASD patient with 76 years old at surgery of observational group had SS of 29.5°, PT of 23.6°, and SPI of 0.8 preoperatively (**A**), the immediate TK, SS and PT post-operatively was 43.3°, 38.8° and 17.5° respectively, and the immediate SPI postoperatively was 0.45 (**B**). She developed PJF with UIV fracture at the 4^th^ month during follow-up

Two representative patients were shown in the Figs. 5 and 6 respectively.

## Discussions

According to those results reported in recent studies [20, 21], full-body balance describes the optimal alignment of the spine in the sagittal plane, resulting from the interaction between the spine and lower limbs, via the pelvis. Moreover, Zhang et al*.* [15] suggested that the pathological hip joints would increase the incidence of proximal junctional failure (PJF) significantly in adult spinal deformity (ASD) patients underwent correction surgery. As a result of, we concluded that the match between spine and hip joints may correlate significantly to the quality of life of ASD patients who have undergone deformity correction surgeries.

In this current study, we proposed the parameter of spinopelvic index (SPI), the ratio of SS to PT, representing the match between spine and hip joints. Comparisons of radiographic parameters between those with and without PJF showed that the SPI had significant differences. According to the best cutoff value of 0.82 for SPI postoperatively, all of those participants were subdivided into the observational (SPI ≤ 0.82) and the control group (SPI > 0.82). The incidence of PJF in the observational group was much higher than that in the control group. Multivariate analysis illustrated that the SPI ≤ 0.82 was associated with increased odds of PJF happening, and the odds ratio was 12.375. As a result of, we concluded that SPI correlated significantly to PJF developing in ASD patients after correction surgery. Additionally, after stratifying all participants by the value of 0.82 (> 0.82 or ≤ 0.82), PJF-free survival time decreased significantly in those with SPI ≤ 0.82, as the representative patient shown in the Fig. 5.

The prevalence of those proximal junctional diseases, such as PJK and PJF, is always very common in ASD after deformity surgical procedures [3, 8, 22]. Radiographic parameters including the lumbar lordosis (LL), pelvic tilt (PT), thoracic kyphosis (TK), pelvic incidence (PI) and sagittal vertical axis (SVA) have been demonstrated to be the significant predictors for PJK or PJF [3, 23–28], which were illustrated the similar results in our current study. Comparisons of radiographic parameters between patients with and without PJF showed that those variables regarding to TK, SS and PI had significant differences. According to the spinopelvic classification proposed by Roussouly et al*.* [7], those suffering from PJF may have pathological full-spinal alignments compared to those without PJF after correction surgery. Moreover, previous studies have been reported that full-spinal realignments in ASD patients correlated strongly to the PJK or PJF developing [8, 9]. However, the results in our current study illustrated that PJF may be resulted from the interaction between spine and hip joints, not just one-single factor. Therefore, we believe that it is necessary to propose the SPI, representing the match between spine and hip joints, to predict PJF developing in ASD patients underwent long-fusion surgery.

In this current study, PI and postoperative SVA were similar between the observational and the control group, however, those individuals in the observational group had much smaller LL and SS, but much larger PT, which illustrated that whole spinopelvic alignments were mildly kyphotic in those with SPI ≤ 0.82. The similar correction in those radiographic parameters including TK, LL, PT and SS would be insufficient for those with SPI ≤ 0.82 who should be performed deformity surgical procedures with combined anterior- posterior approaches [29]. Moreover, those patients may suffer from the mismatch between spine and hip joints, and keep their body inclining forwardly in both sitting and standing positions, which would increase proximal stress and those proximal junctional diseases happened subsequently. As a result of, we believe that the SPI has significantly clinical importance.

Previous studies demonstrated that much larger TK preoperatively was risk factor for PJK [28, 30]. However, Zhang et al*.* [15] suggested that the smaller acetabular anteversion postoperatively would result in PJF developing, which may be accelerated by the larger TK postoperatively. After stratifying all patients by the optimal threshold of 28° for post-TK proposed by professor Zhang et al. [15], post-TK ≥ 28° may decrease significantly the PJF-free survival time, especially for those with SPI ≤ 0.82 (Figs. 4 and 6). Additionally, after multivariate analyzing, we found both post-TK ≥ 28° and SPI ≤ 0.82 had significant association with PJF. The larger TK postoperatively may deteriorate the mismatch between spine and hip joints, which would increase the incidence of PJF in ASD patients after deformity surgical procedures.

Demographic and surgical risk factors for PJK and PJF, involving age, BMI, osteoporosis, the upper instrumented vertebra (UIV), lower instrumented vertebra (LIV), the type of instrumentation and surgical approaches, have been reported [3, 14, 24, 29, 31, 32]. In our current study, comparisons of age, gender and BMI between patients with and without PJF showed no difference, so did that between the observational and the control group. All patients had undergone the surgical procedure of long-fusion (≥ 5) using pedicle screws and 2-rod constructs (titanium alloy) with posterior-only approach, which would rule out those errors caused by surgical materials and operational approaches.

### Limitations

Firstly, the sample size and retrospective research design may undermine the accuracy of those results in our current study. While the impact of the SPI on PJF happening was so significant even in this small series. The presence of osteoporosis for those participants was not recorded due to the missing data of bone mineral density in most patients, which would result in bias that may be decreased by those demographic data regarding to age, gender and BMI although. Lastly, health related questionnaires of life (HRQoL) were not included in this study, which were not involved in the research aims although. Future researches should be needed to establish the relationships between SPI and HRQoL in ASD patients, which may be based on those results in our current study.

## Conclusions

The match between spine and hip joints, namely SPI in our study, correlates significantly to the PJF developing in ASD patients underwent long-fusion surgeries, and should be over 0.82. The incidence of PJF may increase by 12-fold in such individuals with the immediate SPI ≤ 0.82 postoperatively. Moreover, the larger TK postoperatively may deteriorate the mismatch between spine and hip joints, which would accelerate the PJF developing.

## Data Availability

The patients’ data were collected in the affiliated hospital of Shandong University of Traditional Chinese Medicine and the affiliated hospital of Jining medical University. The datasets used and/or analyzed during the current study are available from those corresponding authors on reasonable request. There was no data published previously.

## Abbreviations

ASD: Adult spinal deformity
SPI: Spinopelvic index
PJF: Proximal junctional failure
PJK: Proximal junctional kyphosis
TK: Thoracic kyphosis
LL: Lumbar lordosis
SVA: Sagittal vertical axis
SS: Sacral slope
PT: Pelvic tilt
PI: Pelvic incidence
CI: Confidence interval
UIV: Upper instrumented vertebra
LIV: Lower instrumented vertebra
OR: Odds ratio
